# Painless Tooth Extraction

**Published:** 1885-08-20

**Authors:** 


**Painless Tooth Extraction.**—Dr. Hepburn, in the Independent Practitioner, says that teeth can be extracted without pain in the following manner : The tincture of purified extract of cannabis indica is diluted with from three to five parts of water. This is applied to the gums by rubbing with the finger dampened with the solution. The forceps are also dipped into the solution before applying them to the teeth.—*N. Y. Medical Times.*

